# Comment on “Calmangafodipir Reduces Sensory Alterations and Prevents Intraepidermal Nerve Fibers Loss in a Mouse Model of Oxaliplatin Induced Peripheral Neurotoxicity” *Antioxidants* 2020, *9*, 594

**DOI:** 10.3390/antiox9090802

**Published:** 2020-08-28

**Authors:** Jan Eric Stehr, Ingemar Lundström, Jan Olof G. Karlsson

**Affiliations:** 1 Department of Physics, Chemistry and Biology, Linköping University, 581 83 Linköping, Sweden; ingemar.lundstrom@liu.se; 2 Division of Drug Research/Pharmacology, Linköping University, 581 83 Linköping, Sweden; janolof.karlsson@ktias.com

We have with enthusiasm read the article “Calmangafodipir Reduces Sensory Alterations and Prevents Intraepidermal Nerve Fibers Loss in a Mouse Model of Oxaliplatin Induced Peripheral Neurotoxicity” written by Annalisa Canta, Guido Cavaletti and co-workers and published in Antioxidants [1].

The authors conclude that their study lends mechanistic support that mangafodipir and calmangafodipir exert the neuroprotective effects through their manganese superoxide dismutase (MnSOD)-mimetic and iron chelating activities, in relation to oxaliplatin-associated chemotherapy-induced peripheral neuropathy (CIPN). As authors of a recently published, but by the authors of the current article non-cited, article [2], we would like to make some essential comments. Our article suggests another and, in our opinion, more plausible mechanism behind the efficacy of these compounds, in this particular case, namely that the chelator part of mangafodipir, fodipir or its dephosphorylated metabolite PLED (diPyridoxyL EthylDiamine), binds and increases renal excretion of Pt^2+^ by a process known as chelation therapy. 

Copper (II) (Cu^2+^) binds to fodipir and PLED with an affinity that is more than 1000 times higher than that of Zn^2+^ [3,4]. Similarities between Pt^2+^ and Cu^2+^, with respect to coordinate binding geometry and ionic radius, suggest that Pt^2+^ should bind to fodipir with high affinity [2]. Mainly due to the lack of true water-soluble Pt^2+^ salts, it is, however, not an easy task to determine the affinity of Pt^2+^ for the hexa-dentate chelator fodipir with conventional potentiometry, which is reflected by the apparent absence of formation constants of Pt^2+^ for common hexa-dentate chelators, such as EDTA (Ethylenediaminetetraacetic acid), in the literature. However, by making use of the difference in electron paramagnetic resonance (EPR) spectra of mangafodipir and hexaqua- Mn^2+^ to measure the release of Mn^2+^ from fodipir, as described by Schmidt and co-workers [5], in exchange for Pt^2+^ [from K_2_Pt(Cl)_4_] and Zn^2+^, we demonstrated that Pt^2+^ binds with a statistically significant higher affinity to fodipir compared to Zn^2+^. After administration of mangafodipir into humans, more than 70% is excreted as Zn^2+^-metabolites in the urine within less than 24 hours. To be efficacious as a chelation therapeutic drug it is of crucial importance, as shown, that Pt^2+^ binds with a significantly higher affinity than Zn^2+^ to fodipir or its dephosphorylated metabolite PLED. 

Heavy metal ions bind to vital cellular components, such as structural proteins, enzymes and nucleic acids, and interfere with their functions. Preferred endogenous binding sites for soft and borderline metal ions such as Pt^2+^, Cu^2+^ and Zn^2+^ are thiolates, amides and phenolates [see reference 2]. In the case of oxaliplatin-associated Pt^2+^, it is preferably taken up by organic cation and copper transporters and retained in the dorsal root ganglion (DRG), causing a state of prolonged increase in oxidative and nitrosative stress and consequent nerve degeneration [6].

Although the toxicity and tumoricidal activity of cisplatin differs from that of oxaliplatin, both cause a similar chronic form of CIPN. The differences between them, with respect to therapeutic and toxic activity, are apparently due to distinct differences in pharmacokinetic properties, governed by the organic part (ligands) of them, similar to what is known for other heavy metals. Oxaliplatin has a distribution volume of about 600 L compared to about 20 L for cisplatin [7], implying a much higher lipophilicity of oxaliplatin compared to cisplatin and consequently displays differences in the distribution and toxicity pattern, such as for example the distinct difference in nephrotoxicity between them. However, there is little or no doubt that the underlying causative to chronic CIPN is, in both cases, retention of Pt^2+^.

After administration into patients, oxaliplatin (diaminocyclohexane platinum oxalate) is rapidly metabolized (plasma half-life of less than 30 min) into numerous metabolites and less than 3% of it is converted into the tumoricidal active metabolite diaminocyclohexane platinum dichloride [Pt(dach)Cl_2_] [8] that after sequential replacement of the chlorides with water is a key step in forming a complex with the DNA [9]. It is therefore reasonable to anticipate that it is the non-active Pt^2+^-metabolites that cause oxaliplatin-associated CIPN [8]. Importantly, the pharmacokinetics of Pt^2+^- metabolites of oxaliplatin in plasma is typically triphasic in man, characterized by a short initial distribution phase and a long terminal elimination phase with a half-life of about 11 days [7]. Anticipating that five half-lives are needed to reach the “zero” plasma level, this means that it will take around 55 days to get “all” Pt^2+^ out of the body. Repeated administration of oxaliplatin every 14 days according to the FOLFOX regimen will therefore over time result in an increasing burden of Pt^2+^ and secondary increase in oxidative and nitrosative stress. The long terminal half-life represents a slow release of low molecular weight platinum-conjugates after degradation of cellular macromolecules [7].

Pharmacokinetic data from human volunteers, as reported by Toft et al. [10], show that little or no MnSOD-mimetic active metabolites are present in the blood plasma two hours after administration of mangafodipir, whereas a Zn-metabolite (ZnPLED) is detected within the first 12 hours after administration. PLED has high affinity for Fe^2+/3+^ [3,4], a property together with the MnSOD-mimetic activity that may reduce the secondary increase in cellular stress caused by Pt^2+^. However, such an effect will only be transient and would not fix the ultimate cause of chronic Pt^2+^-associated CIPN, namely retention of Pt^2+^ in the DRG. The fact that Pt^2+^ binds fodipir and PLED with high affinity, as shown in our recent work [2], provides, in our opinion, a more plausible explanation for the therapeutic effects of mangafodipir and calmangafodipir as suggested by Coriat and co-workers [11] and Glimelius and co-workers in patients with chronic oxaliplatin-associated CIPN [12]. The situation in acute cellular stress as those seen in ischemia-reperfusion injury, doxorubicin-induced cardiac toxicity, paracetamol (acetaminophen)-induced liver toxicity and chemotherapy-induced myelosuppression is quite different where the combined MnSOD-mimetic and iron-chelating activity of mangafodipir and calmangafodipir most probably explains the therapeutic effects of these compounds [13].
